# Polymorphisms in the canine monoamine oxidase a (*MAOA*) gene: identification and variation among five broad dog breed groups

**DOI:** 10.1186/s40575-016-0040-2

**Published:** 2017-01-13

**Authors:** James Sacco, Andrew Ruplin, Paul Skonieczny, Michael Ohman

**Affiliations:** Ellis Pharmacogenomics Laboratory, College of Pharmacy and Health Sciences, Drake University, Des Moines, IA 50311 USA

**Keywords:** *MAOA*, Canine, Polymorphism, Breed difference, Aggression

## Abstract

**Background:**

In humans, reduced activity of the enzyme monoamine oxidase type A (MAOA) due to genetic polymorphisms within the *MAOA* gene leads to increased brain neurotransmitter levels associated with aggression. In order to study MAOA genetic diversity in dogs, we designed a preliminary study whose objectives were to identify novel alleles in functionally important regions of the canine *MAOA* gene, and to investigate whether the frequencies of these polymorphisms varied between five broad breed groups (ancient, herding, mastiff, modern European, and mountain). Fifty dogs representing these five breed groups were sequenced.

**Results:**

A total of eleven polymorphisms were found. Seven were single nucleotide polymorphisms (SNPs; two exonic, two intronic and three in the promoter), while four were repeat intronic variations. The most polymorphic loci were repeat regions in introns 1, 2 (7 alleles) and 10 (3 alleles), while the exonic and the promoter regions were highly conserved. Comparison of the allele frequencies of certain microsatellite polymorphisms among the breed groups indicated a decreasing or increasing trend in the number of repeats at different microsatellite loci, as well as the highest genetic diversity for the ancient breeds and the lowest for the most recent mountain breeds, perhaps attributable to canine domestication and recent breed formation. While a specific promoter SNP (−212A > G) is rare in the dog, it is the major allele in wolves. Replacement of this ancestral allele in domestic dogs may lead to the deletion of heat shock factor binding sites on the *MAOA* promoter.

**Conclusions:**

Dogs exhibit significant variation in certain intronic regions of the *MAOA* gene, while the coding and promoter regions are well-conserved. Distinct genetic differences were observed between breed groups. Further studies are now required to establish whether such polymorphisms are associated in any way with MAOA level and canine behaviour including aggression.

**Electronic supplementary material:**

The online version of this article (doi:10.1186/s40575-016-0040-2) contains supplementary material, which is available to authorized users.

## Plain english summary

Canine aggression, whether directed at the owner, strangers, or other dogs, is the most frequent behavioral problem in dogs. Aggressive dogs often end up in shelters where many are euthanized. Aggressive behavior is often due to high levels of neurotransmitters such as dopamine and serotonin in the brain. Recent studies have shown that inherited changes in genes that control neurotransmitter levels may be responsible for aggressive or impulsive behavior in certain dog breeds such as the Belgian Malinois, German Shepherds, and Siberian Huskies. One such gene is called monoamine oxidase A (MAOA), which codes for the MAOA enzyme that degrades neurotransmitters and helps to keep the level of these chemicals constant in the brain, thus preventing unwanted aggression. Variations in the DNA sequence of this gene have been shown to cause increased aggression in people as well as rats. This pilot study explored the genetic diversity of the MAOA gene in fifty dogs, representing 5 broad breed groups (ancient, herding, mastiffs, modern European, and mountain). We discovered 11 novel changes in the DNA sequence of MAOA. The highest number of these DNA changes was observed for ancient breed dogs while the lowest number of changes were observed in more modern breeds such as the mountain dog breed group. Ancient breeds shared a lot of genetic similarity with wolves and coyotes. Other variations were only present in modern breeds such as the mountain breed group. Some of these DNA changes may affect the proper function of the MAOA gene and thus may be responsible for canine aggressive behavior. This research provides valuable information for a follow-up study to discover potential MAOA genetic markers for aggression in dogs. These markers can be used to develop genetic tests for dogs with aggressive behavior so that timely intervention involving behavioral therapy with or without medication can be used to treat the dog. This will reduce the risk for owners to relinquish their dogs to animal shelters.

## Background

Monoamine oxidases (MAO) are enzymes localized on the outer membrane of mitochondria that catalyze the FAD-dependent oxidative deamination of various biogenic amines in the brain as well as in peripheral tissues. Two forms of MAO, designated MAOA and MAOB on the basis of substrate specificity and inhibitor sensitivity, are known to exist. Monoamine oxidase type A (MAOA) preferentially degrades serotonin and dopamine to hydroxyindoleacetic acid (5-HIAA) and homovanillic acid (HVA) respectively [1].

Neurotransmitters can lower the threshold for an aggressive response to environmental stimuli. This has been shown by a number of studies that have implicated MAOA in several behavioral changes in mammals. For example, *MAOA* knockout mice showed increased aggression in adulthood [2], and a rare nonsense mutation in the human *MAOA* gene, which presumably results in low levels of the MAOA enzyme, is associated with impulsive aggression in male humans [3]. Conversely, a landmark study demonstrated that maltreated male children with a genotype conferring high levels of MAOA expression were less likely to develop antisocial problems [4]. This genotype consisted of 5 copies of a variable number tandem repeat (VNTR) in the promoter region [5]. Other studies have shown positive associations between genetic polymorphisms in the human *MAOA* gene and juvenile delinquency [6], impulsivity [7], and female panic and depressive disorders [8, 9]. In general, MAOA enzyme activity varies 50-fold in the population, which is determined in part by genetic polymorphisms [10].

Aggressive behavior is by far the most frequently encountered behavioral problem in dogs. For example, aggression was the presenting complaint in 70% of the 1,644 dogs referred to the Animal Behavior Clinic at Cornell University over a ten year period [11]. Bite injuries have reached epidemic proportions (for example, 45% of children 4 to 18 years old) [12, 13]. Between 17 and 25% of pet dogs relinquished to animal shelters each year are given up because of behavior problems, with substantial numbers of these dogs being euthanized [14, 15]. As is the case with humans, aggression is influenced by both genetic and environmental factors. However, breed-specific behavioral traits such as calmness/aggression are evidence of a large genetic component and specific behaviors show high heritabilities [16, 17]. In addition, aggression is a complex trait regulated by multiple genes.

Naturally-occurring DNA sequence variations, or polymorphisms, in the genes of regulating enzymes, transporters and receptors of the neurotransmitters of the central nervous system have been associated with aggression in dogs. For example, a single copy allele of a variable number tandem repeat (VNTR) and an intronic 12-nucleotide poly (A) insertion in the dopamine transporter gene (*SLC6A3*) are associated with owner-reported seizures, loss of responsiveness to environmental stimuli, episodic aggression, and hyper-vigilance in the Belgian Malinois dog breed [18]. The polymorphism of c.471 T > C in the glutamate transporter (*SLC1A2* or *GLT1*) gene is significantly associated with increased ‘aggression to strangers’ in the Shiba Inu breed [19]. Polymorphisms in the tyrosine hydroxylase and dopamine receptor 4 genes influence impulsivity in German Shepherds and Siberian Huskies [20–22].

Despite that *MAOA* has been shown to be broadly expressed in canine brain [23], and reduced cerebrospinal levels of 5-HIAA and HVA (major metabolites of serotonin and dopamine respectively) were found in aggressive dogs with a history of biting without warning [24], to date there have been no studies on the genetic diversity of the *MAOA* gene among various dog breeds and the potential association of *MAOA* genetic polymorphisms with aggression phenotypes in dogs. As in other mammals, the canine *MAOA* gene is located on the X chromosome, which implies that male dogs, with only one copy of this chromosome, are especially susceptible to high levels of aggression if they have an allele which confers low activity of this enzyme and consequently increased catecholamine levels.

In order to explore the genetic diversity of the *MAOA* gene in dogs, we sequenced functionally important regions of this gene and determine the frequency of novel and existing genetic polymorphisms in pure-breed dogs representing five genetically distinct breed categories.

## Methods

### Animals and sample collection

The fifty unrelated dogs (28 males, 22 females) recruited for this study were either privately owned or provided through the collaboration of Paws & Effect, the Des Moines Kennel Club, and the Des Moines Obedience and Training Club. No dogs were euthanized for the purpose of this study. American Kennel Club certified pure-bred dogs, representing 40 different breeds, were classified in one of five genetically distinct categories: ancient breeds (ANC), herding (HER), mastiff (MAS), modern European (MEU), and mountain (MOU) dogs (Table 1). Cluster analysis has shown that ANC breeds are the lineage most related to wolves, followed by the HER and MAS groups. The most recent canine breed groups are MEU and MOU dogs [25, 26]. None of the dogs in this study exhibited aggressive behavior. Buccal cell samples were collected from the dogs using cheek swabs designed for use in canines (Performagene®, DNA Genotek Inc.), which cause minimal distress to the animals. DNA collected in this manner is stable for at least one year when stored at room temperature. All experimental procedures were approved by the Drake University Institutional Animal Care and Use Committee.

**Table 1:** Breeds used in the study, categorized according to genetic cluster. Each group was represented by 10 dogs

Genetic Cluster	Breeds
Ancient	Akita, Alaskan Malamute (*x*2), Basenji (*x*2), Samoyed (*x*2), Siberian Husky (x3)
Herding	Australian Shepherd Dog, Belgian Malinois, Belgian Tervuren, Border Collie, Cardigan Welsh Corgi, Great Dane, Greyhound, Irish Wolfhound, Rough Collie, Shetland Sheepdog
Mastiff	American Staffordshire Terrier, Boxer, Bulldog, English Mastiff, Labrador Retriever (*x*2), Newfoundland, Pembroke Welsh Corgi, Pitbull Terrier, Yorkshire Terrier
Modern European	Beauceron, Catahoula, Cocker Spaniel, Dalmatian, Doberman Pinscher (*x*2), German Short Haired Pointer, Golden Retriever, Shih Tzu, Toy Poodle
Mountain	German Shepherd (x3), Italian Greyhound, Miniature Pinscher, Rhodesian Ridgeback, Rottweiler, Standard Poodle, St. Bernard, Bernese Mountain Dog

### DNA sequencing

**Fig. 1:**
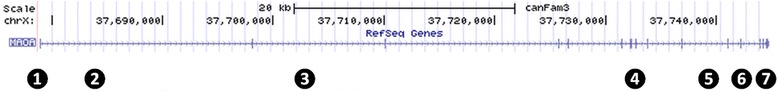
Overall genomic structure and sequencing strategy for the canine *MAOA* gene. Legend: The arrangement of exons is shown relative to the scale provided at the top, which corresponds to the most recent version of the canine genome assembly, canFam3 (available at: https://genome.ucsc.edu/cgi-bin/hgGateway). The position of each of the 7 target regions is indicated by the numbered circles. 1: promoter and exon 1; 2: SINEC_Cf in intron 1; 3: intron 2 repeat region; 4: exons 7 and 8; 5: Intron 10 repeat regions; 6: exon 12; 7: exon 15 and 3′UTR

Following inactivation of nucleases and precipitations of impurities in the buccal samples, genomic DNA was purified via ethanol precipitation. Since the canine *MAOA* gene is very large, spanning more than 66,000 bp, we used the UCSC genome browser to identify, and design polymerase chain reaction (PCR) primers for, specific regions of the gene which are homologous to regions in the human *MAOA* gene for which dysfunctional alleles have been reported, as well as other regions of potential functional importance. This strategy, although not covering the entire gene, enabled us to screen a larger population of dogs, increasing the likelihood of discovering genetic polymorphisms that may ultimately impact the behavioral phenotype of interest. Seven regions of the canine *MAOA* gene were amplified by PCR (Fig. 1). The relevant primer sequences are listed in Additional file 1.

We used 200 ng of DNA in a 25 μl of reaction mixture containing 0.15-0.40 μM of each primer and 12.5 μL of Amplitaq Gold 360 Master Mix (Applied Biosystems, Foster City, CA). After an initial incubation at 95 °C for 10 min, PCR amplification was performed for 40 cycles consisting of 95 °C for 30 s, 60 °C for 30 min and 72 °C for 20-60s (depending on amplicon size), followed by a final extension at 72 °C for 7 min. The specificity of each PCR was checked by electrophoresis on a 1.5% agarose gel. Following purification by Exo-SapIT (Affymetrix, Santa Clara, CA), the amplicons were submitted to bidirectional Sanger sequencing using the Big-Dye Terminator v3.1 (Eurofins Genomics (Louisville, KY). Sequence assembly and identification of genetic polymorphisms was performed using Staden package software (http://staden.sourceforge.net/). Simple repeat sequences and other repeating elements in the canine *MAOA* gene were predicted by the programs Tandem Repeats Finder [27] and RepeatMasker [28]. Comparative genomic analysis between canine and other mammalian *MAOA* sequences was performed using MultiZ [29]. Prediction of any functional effects that these polymorphisms may have on the *MAOA* gene was investigated by web-based software, including LASAGNA [30] for the promoter region, Human Splicing Finder [31] for the intronic regions, and RNAstructure [32] for the transcribed region.

Haploview [33] was used to calculate population genetic descriptors. The chi-squared test for independence was used to assess Hardy–Weinberg equilibrium. Due to the relatively small sample size, a more conservative chi-squared test was used when any expected number was below 5. The polymorphism information content (PIC), which measures the informativeness of a genetic marker for linkage studies, was calculated using PICcalc [34].

To further understand the differences observed in the type and frequency of polymorphisms found between each breed category, the target canine genomic sequences were aligned, using the SRA-BLAST tool (available at https://blast.ncbi.nlm.nih.gov/Blast.cgi), with the homologous sequences in seventeen wolves, five coyotes, and one jackal that are deposited in the sequence read archive (SRA) database (available at: http://www.ncbi.nlm.nih.gov/sra/). Details of the sequences used are given in Additional file 2.

## Results

**Fig. 2:**
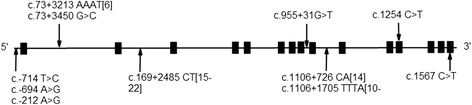
Gene map displaying *MAOA* polymorphisms

A total of 3035 bp, representing portions of two intronic and five exonic coding regions were sequenced. Eleven *MAOA* polymorphisms, consisting of single nucleotide polymorphisms (SNPs) and microsatellite repeats were found (Fig. 2). Eight of these variants were previously unknown. Three SNPs were observed in the promoter region. Multiple alleles were observed for the microsatellite loci in intron 2 (7 alleles) and intron 10 (3 alleles). Two SNPs were found in the coding region studied, c.1254 C > T in exon 12, and c.1567 C > T in exon 15. With the exception of these intron 1 and 10 microsatellite variants, the occurrence of most of the polymorphisms in the dogs studied was low (MAF < 0.1) (Table 2). The observed heterozygosities for the intron 10 variants was lower than expected, suggesting the influence of inbreeding, selection and/or founder effects (Tables 2 and 3). Despite the presence of seven alleles for the intron 2 microsatellite, one allele (CT [19]) was found in 41 out of 50 dogs (74% of dogs were homozygous or hemizygous for this allele), resulting in a low PIC value for this locus (Table 3). Conversely, the intron 10 microsatellite had a higher PIC due to similar allelic frequencies for the three alleles (0.35, 0.44, 0.24 for *n* = 10, 11, 12 respectively) that were found. Both of the microsatellite loci investigated were not in Hardy-Weinberg equilibrium.

**Table 2:** Canine *MAOA* genetic diversity in dogs included in this study

Gene Region	Polymorphism Accession no.	Sequence Variant	H_obs_	H_exp_	HWpval	MAF
Promoter	ss#2019497348	c.-714 T > C	0.000^a^	0.000	1.000	0.04
	ss#2019497349	c.-694 A > G	0.000^a^	0.000	1.000	0.04
	rs85133046	c.-212 A > G	0.000^a^	0.000	1.000	0.03
Intron 1	ss#2019497352	c.73 + 3213 AAAT [6]	0.455	0.496	0.493	0.46
	ss#2019497353	c.73 + 3450 G > C	0.111	0.105	1.000	0.06
Intron 8	rs852643176	c.955 + 31 G > T	0.091	0.087	1.000	0.05
Intron 10	ss#2019497360	c.1106 + 726 CA [14]	0.045	0.127	0.140	0.10
Exon 12	ss#2019497363	c.1254 C > T	0.045	0.044	1.000	0.02
Exon 15	rs852093239	c.1567 C > T	0.091	0.087	1.00	0.05

^a^ Variant found only in male dogs

**Table 3:** Summary data for two canine *MAOA* microsatellites in introns 2 and 10

Microsatellite locus	Accession nos.	Size range	No. of alleles	PIC^a^	H_obs_	H_exp_	HWpval
c.169 + 2485 CT [15–22]	ss#2019497354-8	149–163	7	0.37	0.36	0.39	0.0005
c.1106 + 1705 TTTA [10–12]	ss#2019497361-2	280–288	3	0.52	0.36	0.64	0.002

^a^ Polymorphism Information Content

**Fig. 3:**
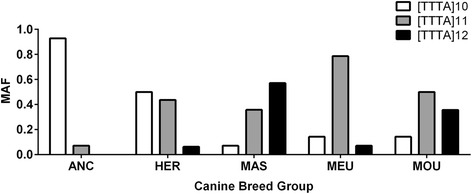
Minor Allele Frequencies (MAF) for the *MAOA* intron 1 TTTA variants across canine breed groups (*n* = 10 for each group)

Dogs which cluster in the ancient breed category were the most genetically distinct and diverse from the other groups, while mountain dogs were the least genetically varied (Table 4). For example, the intron 10 TAAA [10] allele was found in 9 out of 10 ancient breed dogs, while the intron 10 CA [14] allele was present in 6 out of 10 ancient breed dogs and absent in all other dogs (Table 5). The TAAA [11] and TAAA [12] variants were more common in the other breed groups (Fig. 3). The most modern breed group, the mountain dogs, showed the greatest allelic diversity (5 alleles) for the intron 2 microsatellite, while the ancient breeds only had one allele (CT [19]) at this locus.

**Table 4:** Minor allele frequencies for *MAOA* polymorphisms in different breed groups (*n* = 10 for each group)

Variant	ANC	HER	MAS	MEU	MOU
c.-714 T > C	0.077	0.063	0.071	0.000	0.000
c.-694 A > G	0.071	0.063	0.000	0.000	0.000
c.-212 A > G	0.071	0.063	0.000	0.000	0.000
c.73 + 3213 AAAT [6]	0.357	0.500	0.357	0.286	0.429
c.73 + 3450 G > C	0.167	0.071	0.083	0.083	0.000
c.955 + 31 G > T	0.000	0.000	0.143	0.071	0.000
c.1106 + 726 CA [14]	0.500	0.000	0.000	0.000	0.000
c.1254 C > T	0.000	0.063	0.000	0.000	0.000
c.1567 C > T	0.000	0.000	0.143	0.071	0.000

**Table 5:** Minor allele frequencies for the *MAOA* microsatellite alleles in different breed groups (*n* = 10 for each group)

Variant	ANC	HER	MAS	MEU	MOU
c.169 + 2485 CT					
CT [15]	0.000	0.063	0.000	0.000	0.000
CT [17]	0.000	0.063	0.000	0.000	0.071
CT [18]	0.000	0.000	0.000	0.071	0.071
CT [19]	1.000	0.688	0.786	0.714	0.643
CT [20]	0.000	0.188	0.143	0.214	0.071
CT [21]	0.000	0.000	0.000	0.000	0.143
CT [22]	0.000	0.000	0.071	0.000	0.000
c.1106 + 1705 TTTA					
TTTA [10]	0.929	0.500	0.071	0.143	0.143
TTTA [11]	0.071	0.437	0.358	0.786	0.428
TTTA [12]	0.000	0.063	0.571	0.071	0.429

None of the intronic polymorphisms were predicted to have a significant effect on splicing. Similarly, the synonymous coding SNPs were not expected to alter the structure and stability of the *MAOA* transcript. However, the promoter variants were predicted to affect the binding of several transcription factors. The c.-714 T > C variant may result in the deletion of a binding site for E1A binding protein (EP300), as well as generating a new site for the binding of transcription factor 3 (TCF3). The c.-694 A > G polymorphism deletes potential binding sites for the hepatocyte nuclear factor HNF-4 and the nuclear receptor NR2F1 as well as creating a POZ/BTB and AT hook containing zinc finger 1 (PATZ1) binding site. The −212 A > G transition was predicted to generate new sites for heat shock factors (HSF) 1 and 2, as well as a site for spermatogenic leucine zipper 1 (Spz1). MultiZ alignment analysis of the sequence adjacent to the promoter −212 A > G SNP demonstrated that the G allele is conserved in 43 out of 53 mammals, and the A or T alleles are observed in other domesticated species such as cow and pig (Additional file 3).

## Discussion

One way in which genetics may affect a dog’s predisposition to a behavioral disorder such as aggression is by altering the metabolism of neurotransmitters. One important metabolic pathway is oxidative deamination, catalyzed by monoamine oxidase A. Therefore, a study of genetic variation within *MAOA* provides an initial step towards identifying potential functional variants that may be cause changes in gene expression.

Since there is a larger genetic variation between breeds rather than within breeds [35], we decided that specific breeds would be represented by one individual dog whenever possible. This strategy enabled us to discover more genetic polymorphisms than would otherwise be possible.

Although the study investigated represents a small sample size of 50 dogs, eleven polymorphisms were discovered in the target regions. The most polymorphic loci were repeat regions in introns 1, 2 and 10, while the exonic and promoter regions were highly conserved. Reduced levels of heterozygosity were observed for both intron 10 loci, implying that this region has been under recent intense selection.

Distinctive differences were observed in the type and occurrence of *MAOA* genetic variants across broad breed groups. This is expected, since allelic distortion within individual dog breeds makes it possible, via genetic drift or intense artificial selection, for formerly rare variants to become more common in one breed or breed category. The highest genetic diversity within a broad breed group was observed for ancient dog breeds, which is in agreement with recent genome-wide SNP and haplotype analyses [36].

Two synonymous SNVs were found in the coding sequence, one in exon 12, which codes for the functionally important FAD binding site, and the other in exon 15, which codes for the transmembrane region that enables attachment of the protein to the mitochondrial membrane. However, these polymorphisms were not predicted to have any effect either on protein function or transcript stability. No variants were found in exons 1, 7, or 8. SNVs in exon 8 have been associated with Brunner’s disease in humans, a rare recessive X-linked disorder characterized by impulsive aggressiveness and mild mental retardation associated with MAOA deficiency [3, 37]. The exons studied were therefore highly conserved, which is not surprising in view of their importance towards normal MAOA function.

Several studies have demonstrated that a repeat polymorphism in the human *MAOA* promoter, located 1137 base pairs upstream of the start codon [5] has been linked to aggression [6–8]. Initial bioinformatic analysis predicted the existence of a VNTR present as 2.4 copies, as shown in the current canine genome assembly (canFam3). This canine VNTR, which is 90 base pairs long is located 406 base pairs upstream of the translation start site. Therefore, we sequenced the 740 bp sequence upstream of the start codon. Analysis of 70 dogs (the promoter was sequenced in an additional 20 dogs, data not shown) did not indicate any repeat variation at this locus, a result confirmed by a recent study [38]. Indeed, the canine *MAOA* proximal promoter region appears to be relatively well-conserved in dogs. Three polymorphisms were found in the promoter regions: c.-714 C > T, c.-694 A > G, c.-212 A > G. The c.-212A > G transition, located in the 5′ untranslated region, was only found in two male dogs, an Alaskan Malamute (ANC) and a Belgian Malinois (HER), and was predicted to lead to the formation of two new heat shock factor (HSF) binding sites. These transcription factors are involved in the inducible expression of a wide variety of genes [39]. Using comparative genomics tools we found that the variant G allele appears to be the wild-type allele in wolves, coyotes, and other carnivores such as the giant panda and ferret, as well as less related mammals such as humans and dolphins. The A or T allele also appears to be the major allele in other domesticated species, including cow, goat, and pig (for sequence alignments, refer to Additional file 3). Therefore, it appears that the ancestral G allele was replaced by the A allele in the course of domestication of the dog, in the process potentially removing an HSF binding site. Whether this change is functionally significant and is related to domestication, perhaps by increasing MAOA expression in dogs relative to their wild ancestors and thus increasing docility, remains to be investigated.

Another target DNA sequence was a canine-specific short interspersed nuclear element (SINEC_Cf), located in *MAOA* intron 1. These aberrantly inserted DNA sequences are degenerate retrotransposons of the lysine t-RNA class, which are frequently located in positions affecting gene expression and have been proposed as a source of canine phenotypic diversity [40]. Insertion of SINEC_Cf elements in the hypocretin receptor-2 and PTPLA genes cause canine narcolepsy in Doberman pinschers and centronuclear myopathy in Labrador retrievers respectively [41, 42]. The SINEC-Cf was found in all the dogs studied as well as in the available wolf and coyote sequences, implying that this must be an ancestral polymorphism rather than a recent derived mutation. An ATTT insertion that was observed in 22 out of 50 dogs was located within a repeat sequence between a CT repeat region and the poly-A tail that is typical of such elements. Whereas this ATTT [6] allele occurred at a MAF of 0.3-0.5 in the dogs studied, it appears to be absent or uncommon in wolves and coyotes, in which the ATTT [4] and ATTT [5] alleles predominate.

Microsatellites are stretches of tandemly repeated sequences of short sequence motifs of 6 or fewer nucleotides. Dinucleotide repeat variants in intron 2 of the human *MAOA* gene have been associated with female bipolar disorder [43]. A similar microsatellite was predicted to be present in intron 2 of the canine *MAOA* gene. Seven different alleles, consisting of variable numbers of CT repeats ranging from 15 to 22, were observed at a microsatellite locus in intron 2. This is similar to the human locus, for which eight alleles have been [43]. While the most common repeat number was 19, this was the only allele observed in ANC dogs and in the available wolf and coyote sequences, while the other, more recent, dog breed groups all had three alleles or more. This may indicate that recent breed formation is increasing the number of repeats at this locus.

This phenomenon of increasing repeats with domestication was also evident at a microsatellite TTTA repeat in intron 10, first reported by Klukowska and co-workers [44]. While ANC dogs, wolves, coyotes and foxes have 10 repeats or less, the occurrence of the TTTA [11] and TTTA [12] alleles increases in the herding dogs, the next oldest breed group. These two alleles predominate in the three other, most recent breed groups (MAS, MEU and MOU). On the other hand, it appears that the number of CA repeats in a microsatellite located a few hundred bases upstream in the same intron appears to be decreasing with domestication, since while the CA [13] allele was ubiquitous in four breed groups, 6 out of 10 ANC dogs had the CA [14] allele. The evidence from wolves is in agreement with this conclusion since their genetic sequences contains from 14 up to 18 repeats at this locus. Both this tetranucleotide repeat and the dinucleotide intron 2 repeat are larger than 18 bp and may be subject to more frequent replication slippage and hence exhibit higher mutation rates [45] resulting in an increased number of repeat length variants. As a matter of fact, this increased mutation rate is a genome-wide phenomenon in the dog and may be one of the factors responsible for the rapid evolutionary change resulting in widespread phenotypic diversity [46].

Although we designed the study to focus on broad canine breed groups in order to discover as many variants as possible, this approach limited the estimation of allelic frequencies and meaningful haplotypes for individual breeds, as well as the calculation of linkage disequilibrium between the polymorphisms found. In order to address this limitation, a follow-up to this study will be to genotype more dog breeds and include more dogs representing specific breeds.

## Conclusions

Dogs exhibit significant variation in certain intronic regions of the *MAOA* gene, while other areas such as the coding and the proximal promoter regions are well-conserved. The *MAOA* sequences in the ancient dog breeds were the most divergent, and for some loci, the most similar to wolves. Less diversity was observed for more modern breed groups, in particular mountain breed dogs. An important finding of this study was that a decreasing or increasing trend in the number of repeats at different microsatellite loci may be related to canine domestication and recent breed formation. The ultimate aim of this research will be to investigate whether these *MAOA* polymorphisms are over-represented in dogs with aggressive phenotypes.

## Additional files

Additional file 1: Canine *MAOA* target regions and primer sequences (5′ → 3′) used in this study. (DOCX 12 kb)
Additional file 2: Wolf, coyote and golden jackal sequence read archive (SRA) sequences used to compare with homologous sequences in domestic dogs. (DOCX 12 kb)
Additional file 3: MultiZ Alignment of -212G > A polymorphism (shown in uppercase) locus in 53 mammalian species. The dog sequence is highlighted in yellow. The sequences from the other canids (wolf, coyote, Golden jackal) were obtained from the sequence read archive (SRA) database. (DOCX 19 kb)
