# Supplementary material for: Polymorphisms in the canine monoamine oxidase a (MAOA) gene: identification and variation among five broad dog breed groups
Source: Canine Genet Epidemiol. 2017 Jan 13;4:1. doi: 10.1186/s40575-016-0040-2 (PMC5237129; doi:10.1186/s40575-016-0040-2)
Supplement: Additional file 1: — Canine MAOA target regions and primer sequences (5′ → 3′) used in this study. (DOCX 12 kb) [file 40575_2016_40_MOESM1_ESM.docx]

**Additional File 1.** Canine *MAOA* target regions and primer sequences (5`→3`) used in this study

| ***MAOA* Target region** | **Forward Primer** | **Reverse Primer** |
| --- | --- | --- |
| Proximal Promoter and Exon 1 | GTGCAAGGACCACCTACACC | CCACTACGGTCCACACTGAC |
| Intron 1 SINEC_cf | GCATCGAGTCCTCTGCCTAT | CCAAGTCCTTCTTGAAACTGC |
| Intron 2 CT repeat | TGGAGACCCTGGATGAGTTC | GCATGCCGTTTTCATTTTG |
| Exons 7 and 8 | CCTGTGTTTTGCTTTCTGACC | TGAGATTTCTGGCCCTTTCT |
| Intron 10 CA repeat | TTCTGGGCTTCCTTTCTTGA | TGCATCCCAAAGTCATCAGA |
| Intron 10 TTTA repeat | GGGAAGAAGGGATGGAGAAG | TCCCTCTCCCTCTGTCACTCT |
| Exon 12 | TTAAGGTAAAATATACGAGAGCCTTC | TCGCTTATGGGTTTAAGGTCA |
| Exon 15 | TGACATGGCAGACGTGTTCT | CCAGGCAAATAACATGATGG |
