# Supplementary material for: Polymorphisms in the canine monoamine oxidase a (MAOA) gene: identification and variation among five broad dog breed groups
Source: Canine Genet Epidemiol. 2017 Jan 13;4:1. doi: 10.1186/s40575-016-0040-2 (PMC5237129; doi:10.1186/s40575-016-0040-2)
Supplement: Additional file 2: — Wolf, coyote and golden jackal sequence read archive (SRA) sequences used to compare with homologous sequences in domestic dogs. (DOCX 12 kb) [file 40575_2016_40_MOESM2_ESM.docx]

**Additional File 2.** Wolf, coyote and golden jackal sequence read archive (SRA) sequences used to compare with homologous sequences in domestic dogs.

| **Population** | **n** | **SRA** | **Species** | **Sub-group** |
| --- | --- | --- | --- | --- |
| Great Lakes (Northern Minnesota), USA | 1 | SRS661486 | Grey Wolf | Grey Wolf |
| Iberia, Spain | 1 | SRS661495 | Grey Wolf | Grey Wolf |
| India (lowland wolf) | 1 | SRS661487 | Grey Wolf | Grey Wolf |
| Iran | 1 | SRS661488 | Grey Wolf | Grey Wolf |
| Portugal (Northern Portugal) | 1 | SRS661492 | Grey Wolf | Grey Wolf |
| Yellowstone National Park, USA | 3 | SRS661496 SRS661497 SRS661498 | Grey Wolf | Grey Wolf |
| USA | 2 | SRS661493 SRS661494 | Grey Wolf | Red Wolf |
| Mexico | 2 | SRS661490  SRS661491 | Grey Wolf | Grey Wolf |
| Italy | 1 | SRS661489 | Grey Wolf | Grey Wolf |
| Alaska | 1 | SRS1589013 | Grey Wolf | Grey Wolf |
| China | 1 | SRS1025418 | Grey Wolf | Grey Wolf |
| Croatia | 1 | SRS1025420 | Grey Wolf | Grey Wolf |
| Israel | 1 | SRS984773 | Grey Wolf | Grey Wolf |
| Coyote Midwest | 1 | SRS661479 | Canis latrans |  |
| Coyote Alabama | 1 | SRS661478 | Canis latrans |  |
| Coyote California | 3 | SRS661477 | Canis latrans |  |
| Golden Jackal | 1 | SRS1025419 | Canis aureus |  |
