# Supplementary material for: Polymorphisms in the canine monoamine oxidase a (MAOA) gene: identification and variation among five broad dog breed groups
Source: Canine Genet Epidemiol. 2017 Jan 13;4:1. doi: 10.1186/s40575-016-0040-2 (PMC5237129; doi:10.1186/s40575-016-0040-2)
Supplement: Additional file 3: — MultiZ Alignment of -212G > A polymorphism (shown in uppercase) locus in 53 mammalian species. The dog sequence is highlighted in yellow. The sequences from the other canids (wolf, coyote, Golden jackal) were obtained from the sequence read archive (SRA) database. (DOCX 19 kb) [file 40575_2016_40_MOESM3_ESM.docx]

**Additional File 3**. MultiZ Alignment of -212G>A polymorphism (shown in uppercase) locus in 53 mammalian species. The dog sequence is highlighted in yellow. The sequences from the other canids (wolf, coyote, Golden jackal) were obtained from the sequence read archive (SRA) database.

**Alignment block 3 of 4 in window, 43656121 - 43656145, 25 bps**

[B](https://genome.ucsc.edu/cgi-bin/hgTracks?db=hg38&ct=&position=chrX%3A43656121-43656145) [D](https://genome.ucsc.edu/cgi-bin/hgc?o=43656120&g=getDna&i=chrX&c=chrX&l=43656120&r=43656145&db=hg38) Human gctcccccc**G**ggtatcagctga---aac

[B](https://genome.ucsc.edu/cgi-bin/hgTracks?db=panTro4&ct=&position=chrX%3A44101749-44101773) [D](https://genome.ucsc.edu/cgi-bin/hgc?o=44101748&g=getDna&i=chrX&c=chrX&l=44101748&r=44101773&db=panTro4) Chimp gctcccccc**G**ggtatcagctga---aac

[B](https://genome.ucsc.edu/cgi-bin/hgTracks?db=gorGor3&ct=&position=chrX%3A44354182-44354206) [D](https://genome.ucsc.edu/cgi-bin/hgc?o=44354181&g=getDna&i=chrX&c=chrX&l=44354181&r=44354206&db=gorGor3) Gorilla gctcccccc**G**ggtatcagctga---aac

[B](https://genome.ucsc.edu/cgi-bin/hgTracks?db=ponAbe2&ct=&position=chrX%3A44280911-44280935) [D](https://genome.ucsc.edu/cgi-bin/hgc?o=44280910&g=getDna&i=chrX&c=chrX&l=44280910&r=44280935&db=ponAbe2) Orangutan gctcccccc**G**ggtatcagctga---aac

[B](https://genome.ucsc.edu/cgi-bin/hgTracks?db=nomLeu3&ct=&position=chrX%3A48231196-48231220) [D](https://genome.ucsc.edu/cgi-bin/hgc?o=48231195&g=getDna&i=chrX&c=chrX&l=48231195&r=48231220&db=nomLeu3&hgSeq.revComp=on) Gibbon gctcccccc**G**ggtatcagctga---aag

[B](https://genome.ucsc.edu/cgi-bin/hgTracks?db=rheMac3&ct=&position=chrX%3A42398599-42398623) [D](https://genome.ucsc.edu/cgi-bin/hgc?o=42398598&g=getDna&i=chrX&c=chrX&l=42398598&r=42398623&db=rheMac3) Rhesus gctcccccc**G**ggtatcagctga---aag

[B](https://genome.ucsc.edu/cgi-bin/hgTracks?db=macFas5&ct=&position=chrUn_KE145957%3A25661-25685) [D](https://genome.ucsc.edu/cgi-bin/hgc?o=25660&g=getDna&i=chrUn_KE145957&c=chrUn_KE145957&l=25660&r=25685&db=macFas5) Crab-eating macaque gctcccccc**G**ggtatcagctga---aag

[B](https://genome.ucsc.edu/cgi-bin/hgTracks?db=papAnu2&ct=&position=JH685061%3A23237-23261) [D](https://genome.ucsc.edu/cgi-bin/hgc?o=23236&g=getDna&i=JH685061&c=JH685061&l=23236&r=23261&db=papAnu2) Baboon gctcccccc**G**ggtatcagctga---aag

Green monkey gctcccccc**G**ggtatcagctga---aag

[B](https://genome.ucsc.edu/cgi-bin/hgTracks?db=calJac3&ct=&position=chrX%3A42627973-42627997) [D](https://genome.ucsc.edu/cgi-bin/hgc?o=42627972&g=getDna&i=chrX&c=chrX&l=42627972&r=42627997&db=calJac3) Marmoset gctcccgcc**G**ggcatcagctga---aaa

[B](https://genome.ucsc.edu/cgi-bin/hgTracks?db=saiBol1&ct=&position=JH378220%3A926-950) [D](https://genome.ucsc.edu/cgi-bin/hgc?o=925&g=getDna&i=JH378220&c=JH378220&l=925&r=950&db=saiBol1) Squirrel monkey gctcccgcc**G**ggcatcagcaga---aag

[B](https://genome.ucsc.edu/cgi-bin/hgTracks?db=otoGar3&ct=&position=GL873711%3A2769589-2769613) [D](https://genome.ucsc.edu/cgi-bin/hgc?o=2769588&g=getDna&i=GL873711&c=GL873711&l=2769588&r=2769613&db=otoGar3) Bushbaby ggtcccgcc**G**ggtatcagctgg---agg

Chinese tree shrew cctccctcc**C**ggtatctctaga---agg

[B](https://genome.ucsc.edu/cgi-bin/hgTracks?db=speTri2&ct=&position=JH393312%3A9816505-9816526) [D](https://genome.ucsc.edu/cgi-bin/hgc?o=9816504&g=getDna&i=JH393312&c=JH393312&l=9816504&r=9816526&db=speTri2) Squirrel gctccccgc**T**ggtatctctgga------

Lesser Egyptian jerboa gctgtcctt**G**ggtatctctaga------

Prairie vole gctctccct**G**ggtttctcagct------

Golden hamster gctctccct**G**ggtatctcagca------

[B](https://genome.ucsc.edu/cgi-bin/hgTracks?db=mm10&ct=&position=chrX%3A16619620-16619641) [D](https://genome.ucsc.edu/cgi-bin/hgc?o=16619619&g=getDna&i=chrX&c=chrX&l=16619619&r=16619641&db=mm10) Mouse gctctccct**G**ggtatctcagca------

[B](https://genome.ucsc.edu/cgi-bin/hgTracks?db=rn6&ct=&position=chrX%3A6620886-6620907) [D](https://genome.ucsc.edu/cgi-bin/hgc?o=6620885&g=getDna&i=chrX&c=chrX&l=6620885&r=6620907&db=rn6&hgSeq.revComp=on) Rat gctctccct**G**ggtatctcagca------

[B](https://genome.ucsc.edu/cgi-bin/hgTracks?db=hetGla2&ct=&position=JH602075%3A11820664-11820685) [D](https://genome.ucsc.edu/cgi-bin/hgc?o=11820663&g=getDna&i=JH602075&c=JH602075&l=11820663&r=11820685&db=hetGla2&hgSeq.revComp=on) Naked mole-rat gcttccccg**G**gggagctctagc------

[B](https://genome.ucsc.edu/cgi-bin/hgTracks?db=cavPor3&ct=&position=scaffold_33%3A14713854-14713875) [D](https://genome.ucsc.edu/cgi-bin/hgc?o=14713853&g=getDna&i=scaffold_33&c=scaffold_33&l=14713853&r=14713875&db=cavPor3&hgSeq.revComp=on) Guinea pig gcttcgccc**G**gggagctaaacc------

Chinchilla gctcccccg**G**ggcatctatggg------

Brush-tailed rat gcttccccg**G**gggagctctacc------

[B](https://genome.ucsc.edu/cgi-bin/hgTracks?db=oryCun2&ct=&position=chrX%3A29088483-29088507) [D](https://genome.ucsc.edu/cgi-bin/hgc?o=29088482&g=getDna&i=chrX&c=chrX&l=29088482&r=29088507&db=oryCun2) Rabbit gctccaccc**G**ggtatctctagaagg---

[B](https://genome.ucsc.edu/cgi-bin/hgTracks?db=ochPri3&ct=&position=JH802090%3A19655391-19655415) [D](https://genome.ucsc.edu/cgi-bin/hgc?o=19655390&g=getDna&i=JH802090&c=JH802090&l=19655390&r=19655415&db=ochPri3) Pika gctgcaacc**G**cctatctctagaagg---

[B](https://genome.ucsc.edu/cgi-bin/hgTracks?db=susScr3&ct=&position=chrX%3A43208849-43208873) [D](https://genome.ucsc.edu/cgi-bin/hgc?o=43208848&g=getDna&i=chrX&c=chrX&l=43208848&r=43208873&db=susScr3&hgSeq.revComp=on) Pig gttaccccc**T**aacagcggtgga---agg

[B](https://genome.ucsc.edu/cgi-bin/hgTracks?db=vicPac2&ct=&position=KB632607%3A44289-44313) [D](https://genome.ucsc.edu/cgi-bin/hgc?o=44288&g=getDna&i=KB632607&c=KB632607&l=44288&r=44313&db=vicPac2) Alpaca gtagtcccc**G**aacatcggtaga---agg

[B](https://genome.ucsc.edu/cgi-bin/hgTracks?db=turTru2&ct=&position=JH472536%3A408999-409023) [D](https://genome.ucsc.edu/cgi-bin/hgc?o=408998&g=getDna&i=JH472536&c=JH472536&l=408998&r=409023&db=turTru2&hgSeq.revComp=on) Dolphin gttaccccc**G**aacatcggtgga---aga

Killer whale gttaccccc**G**aacatcggtgga---aga

Tibetan antelope gttaccccg**A**aagattagtaga---agg

[B](https://genome.ucsc.edu/cgi-bin/hgTracks?db=bosTau8&ct=&position=chrX%3A105462548-105462572) [D](https://genome.ucsc.edu/cgi-bin/hgc?o=105462547&g=getDna&i=chrX&c=chrX&l=105462547&r=105462572&db=bosTau8&hgSeq.revComp=on) Cow gttaccccc**A**aagattagtgga---agg

[B](https://genome.ucsc.edu/cgi-bin/hgTracks?db=oviAri3&ct=&position=chrX%3A40680161-40680185) [D](https://genome.ucsc.edu/cgi-bin/hgc?o=40680160&g=getDna&i=chrX&c=chrX&l=40680160&r=40680185&db=oviAri3) Sheep gttaccccg**A**aagattagtaga---agg

Domestic goat gttaccccg**A**aagattagtaga---agg

[B](https://genome.ucsc.edu/cgi-bin/hgTracks?db=equCab2&ct=&position=chrX%3A35379679-35379703) [D](https://genome.ucsc.edu/cgi-bin/hgc?o=35379678&g=getDna&i=chrX&c=chrX&l=35379678&r=35379703&db=equCab2) Horse gctcctccc**T**ggattcggtact---agg

[B](https://genome.ucsc.edu/cgi-bin/hgTracks?db=cerSim1&ct=&position=JH767773%3A6022332-6022356) [D](https://genome.ucsc.edu/cgi-bin/hgc?o=6022331&g=getDna&i=JH767773&c=JH767773&l=6022331&r=6022356&db=cerSim1&hgSeq.revComp=on) White rhinoceros gcgccccct**G**gagttcgctaca---aag

[B](https://genome.ucsc.edu/cgi-bin/hgTracks?db=felCat8&ct=&position=chrX%3A38258532-38258556) [D](https://genome.ucsc.edu/cgi-bin/hgc?o=38258531&g=getDna&i=chrX&c=chrX&l=38258531&r=38258556&db=felCat8) Cat gctcccccc**G**gggttcgcttca---agg

[B](https://genome.ucsc.edu/cgi-bin/hgTracks?db=canFam3&ct=&position=chrX%3A37678712-37678736) [D](https://genome.ucsc.edu/cgi-bin/hgc?o=37678711&g=getDna&i=chrX&c=chrX&l=37678711&r=37678736&db=canFam3) **Dog gtaccccccAaacatccgtgga---agg**

Wolf gtacccccc**G**aacatccgtgga---agg

Coyote gtacccccc**G**aacatccgtgga---agg

Golden jackal gtacccccc**G**aacatccgtgga---agg

[B](https://genome.ucsc.edu/cgi-bin/hgTracks?db=musFur1&ct=&position=GL896945%3A6389534-6389558) [D](https://genome.ucsc.edu/cgi-bin/hgc?o=6389533&g=getDna&i=GL896945&c=GL896945&l=6389533&r=6389558&db=musFur1) Ferret gtgcccccc**G**aacatccgcgga---agg

[B](https://genome.ucsc.edu/cgi-bin/hgTracks?db=ailMel1&ct=&position=GL192969.1%3A220399-220423) [D](https://genome.ucsc.edu/cgi-bin/hgc?o=220398&g=getDna&i=GL192969.1&c=GL192969.1&l=220398&r=220423&db=ailMel1&hgSeq.revComp=on) Panda gtgcccccc**G**aacatccgcgga---agg

Pacific walrus gtgcccccc**G**aacatccgcgga---agg

Weddell seal gtgcccccc**G**aacatccacgga---agg

Black flying-fox gtacgcccc**G**aacatccgtgga---agg

[B](https://genome.ucsc.edu/cgi-bin/hgTracks?db=pteVam1&ct=&position=scaffold_4755%3A7354-7378) [D](https://genome.ucsc.edu/cgi-bin/hgc?o=7353&g=getDna&i=scaffold_4755&c=scaffold_4755&l=7353&r=7378&db=pteVam1) Megabat gtacgcccc**G**aacatccgtgga---agg

Big brown bat gtaccagtc**G**aatgcccgtgga---ggc

David's myotis (bat) gtgtcagtc**G**aatgccaatgga---agc

[B](https://genome.ucsc.edu/cgi-bin/hgTracks?db=myoLuc2&ct=&position=GL429843%3A3413504-3413528) [D](https://genome.ucsc.edu/cgi-bin/hgc?o=3413503&g=getDna&i=GL429843&c=GL429843&l=3413503&r=3413528&db=myoLuc2) Microbat gtgtcagtc**G**aatgccaatgga---agc

[B](https://genome.ucsc.edu/cgi-bin/hgTracks?db=eriEur2&ct=&position=JH835654%3A1567764-1567788) [D](https://genome.ucsc.edu/cgi-bin/hgc?o=1567763&g=getDna&i=JH835654&c=JH835654&l=1567763&r=1567788&db=eriEur2&hgSeq.revComp=on) Hedgehog aacttctgg**G**taaatctacggt---agg

Star-nosed mole gttctccca**C**agtgtctgtggg---agg

[B](https://genome.ucsc.edu/cgi-bin/hgTracks?db=loxAfr3&ct=&position=scaffold_56%3A8561969-8561993) [D](https://genome.ucsc.edu/cgi-bin/hgc?o=8561968&g=getDna&i=scaffold_56&c=scaffold_56&l=8561968&r=8561993&db=loxAfr3&hgSeq.revComp=on) Elephant gctccccca**G**ggtatccgtaca---agg

[B](https://genome.ucsc.edu/cgi-bin/hgTracks?db=triMan1&ct=&position=JH594652%3A10429423-10429447) [D](https://genome.ucsc.edu/cgi-bin/hgc?o=10429422&g=getDna&i=JH594652&c=JH594652&l=10429422&r=10429447&db=triMan1&hgSeq.revComp=on) Manatee gctctcccc**G**ggtatctgtatc---aag

Cape golden mole gctctccca**G**ggtatcagtatc---aag

Aardvark gctcccccc**G**agtatccgtaca---agg

[B](https://genome.ucsc.edu/cgi-bin/hgTracks?db=dasNov3&ct=&position=JH566161%3A135925-135949) [D](https://genome.ucsc.edu/cgi-bin/hgc?o=135924&g=getDna&i=JH566161&c=JH566161&l=135924&r=135949&db=dasNov3&hgSeq.revComp=on) Armadillo gctgccccc**G**ggattcggtaac---agc

[B](https://genome.ucsc.edu/cgi-bin/hgTracks?db=sorAra2&ct=&position=JH798205%3A5909559-5909646) [D](https://genome.ucsc.edu/cgi-bin/hgc?o=5909558&g=getDna&i=JH798205&c=JH798205&l=5909558&r=5909646&db=sorAra2&hgSeq.revComp=on) Shrew ============================

Bactrian camel NNNNNNNNNNNNNNNNNNNNNNNNNNNN
